# Exploring the effects of anodal and cathodal high definition transcranial direct current stimulation targeting the dorsal anterior cingulate cortex

**DOI:** 10.1038/s41598-018-22730-x

**Published:** 2018-03-13

**Authors:** Wing Ting To, Justin Eroh, John Hart, Sven Vanneste

**Affiliations:** The University of Texas at Dallas, School of Behavioral and Brain Sciences, 800 West Campbell Road, Texas, 75080 USA

## Abstract

The dorsal anterior cingulate cortex (dACC) has been identified as a core region affected by many disorders, representing a promising target for neuromodulation. High Definition-transcranial Direct Current Stimulation (HD-tDCS) is a non-invasive neuromodulation technique that has already shown promising outcomes and has been tested to engage deeper structures. This study investigates whether it is possible to modulate dACC activity using anodal and cathodal HD-tDCS. Furthermore, it examines what effects anodal and cathodal HD-tDCS targeting dACC have on cognitive and emotional processing. Forty-five healthy subjects were randomly assigned to 1 of 3 groups: anodal, cathodal, and sham. Resting-state electroencephalography (rsEEG) and a cognitive and emotional Counting Stroop task were administered before and after HD-tDCS. RsEEG showed changes: anodal HD-tDCS showed significant increase in beta frequency band activity in dACC, while cathodal HD-tDCS led to significant increase in activity at dorsal and rostral ACC in the theta frequency band. Behavioral changes were also found after anodal HD-tDCS in the cognitive Counting Stroop for incongruent trials and after cathodal HD-tDCS in the emotional Counting Stroop for emotional trials. This study demonstrated that HD-tDCS is able to modulate dACC activity, suggesting that it has the potential to be used as a treatment tool.

## Introduction

The anterior cingulate cortex has been demonstrated to be an important brain region in psychopathology as neuroimaging studies have shown dysfunctional anterior cingulate cortex activity in many neuropsychiatric disorders^1^. The success of bilateral cingulotomy psychosurgery in relieving psychiatric symptoms further supports the role of the dorsal anterior cingulate cortex in the regulation of emotional behavior^1^. Recently, a subregion of the anterior cingulate cortex more specifically the dorsal anterior cingulate cortex (dACC), a node in the ‘salience’ network, has been identified to be a core region affected across most psychiatric disorders, representing a promising target for invasive and non-invasive neuromodulation^2,3^.

In the past decades, invasive and non-invasive neuromodulation have been investigated to target deeper brain areas, such as the dACC. These techniques are interesting as they can extend the investigation of brain functions by disrupting or normalizing neural activity transiently^4–6^. Invasive brain stimulation techniques targeting the dACC, such as deep brain stimulation, have been investigated in patients with pain^7–10^, tinnitus^11^, alcohol addiction^12^, and obsessive-compulsive disorder^13^ and have resulted in relevant symptom reduction. With the development of more innovative brain stimulation techniques, non-invasive brain stimulation such as double-cone coil (DCC) repetitive transcranial magnetic stimulation (rTMS) has demonstrated the capability to target deeper brain areas (depths of approximately 4 cm), such as the dACC, as a research tool in healthy subjects and as a treatment tool in patient populations. Hayward and colleagues (2004, 2007) and Harmer and colleagues (2001) have indicated that they were able to target the dACC with DCC rTMS to investigate the role of the dACC in cognitive, affective, and emotional processing in healthy subjects^4,14,15^. DCC rTMS targeting the dACC has also demonstrated its use in treating various disorders including depression^16^, obsessive-compulsive disorder^17,18^, alcohol addiction^12,19^, and tinnitus^11,20,21^ and in investigating pain in patients with chronic disorders of consciousness^22^. Studies have also examined other rTMS coil approaches to target deeper structures^23–25^. Although rTMS is considered an important investigational and treatment tool to target the dACC and other deeper structures, it has some adverse effects, including possible induction of seizure, headache, local pain, and discomfort^26,27^.

Researchers are lately moving towards safer, non-invasive neuromodulation approaches using electrical stimulation, such as transcranial direct current stimulation (tDCS). TDCS uses continuous electrical current flowing from one electrode serving as the anode to another electrode serving as the cathode to modulate an area of interest^28^. The effect of tDCS depends on the polarity of the stimulation. The simplest account for the effects of tDCS assumes a region of “increased excitability” in the cortex directly under the anodal electrode, and a region of “decreased excitability” under the cathode electrode^29–31^, with comparable proposed effects on the underlying cortices and their operations, such that anodal tDCS improves and cathodal tDCS worsens cognitive and behavioral functions supported by these regions^32,33^. However, the translation of anodal and cathodal tDCS into a ‘facilitation’ (anodal = facilitation) or ‘inhibition’ (cathodal = inhibition) in cognitive and behavioral functions is often more complicated^33–35^. More recently, the technique of High Definition tDCS (HD-tDCS) has been introduced using arrays of smaller “high definition” electrodes to enable more complex (i.e. using multiple electrodes to set up a variation of montages to guide current flow, e.g. 4 × 1 electrode set up, 2 × 2 electrode set up,…)^36^, and focal stimulation procedures^37–42^. Although studies have been examining HD-tDCS as a potential treatment for various disorders (e.g. refs^36,43–45^), more research is needed to determine whether HD-tDCS can target deeper brain structures. As the dACC is an important target for the treatment of different neurological and neuropsychiatric disorders, it is particularly important to explore whether HD-tDCS is capable of modulating this brain area.

To assess the function of the dACC and possible changes due to non-invasive neuromodulation, studies have frequently used the Stroop task since it is one of the most well-studied cognitive paradigms for investigating the anterior cingulate cortex, amongst other brain areas^1,46^. Hayward and colleagues (2004, 2007) have used an adjusted computerized version of the Stroop task, namely a cognitive Counting Stroop task, to examine whether DCC rTMS is capable of modulating the dACC^14,15^. Research had previously demonstrated that the cognitive Counting Stroop task engages the dACC^47,48^, whereas the emotional Counting Stroop task is supported by the rostral/ventral anterior cingulate cortex in healthy subjects^47,49^. However, the role of the dACC in emotional processing remains unclear because the emotional Counting Stroop task was not administered in these studies. The dACC has deliberately been targeted using lesioning techniques (i.e. cingulotomy psychosurgery) and neuromodulation techniques (e.g. deep brain stimulation, TMS) for the treatment of various disorders, including obsessive-compulsive disorders, pain, addiction, and tinnitus (e.g. refs^7,11–13,19,23,50,51^). These findings point to a more general role of the dACC than involvement in cognitive processing alone. By using an adjusted Counting Stroop task that includes both cognitive and emotional components of the task, it becomes possible to assess the potential role of the dACC in cognitive and emotional processing by viewing the behavioral changes in these tasks following HD-tDCS. Other assessments were included in the study to control for other effects, such as anxiety (State-Trait Anxiety Inventory), general attention, and executive functions (Trail Making Tests A and B).

The objective of this study is to explore the effects of HD-tDCS targeting the dACC. First, we want to examine whether we can affect dACC activity using anodal and cathodal HD-tDCS measured via resting-state Electroencephalography (EEG). We hypothesize that HD-tDCS is able to modify dACC activity using anodal and cathodal stimulation. Second, we aim to investigate what effect anodal and cathodal HD-tDCS targeting the dACC have on cognitive and emotional processing using a cognitive and emotional Counting Stroop task. We hypothesize that anodal HD-tDCS will result in a general (cognitive and emotional) improvement (i.e. facilitation) in the cognitive and emotional Counting Stroop task. Regarding cathodal HD-tDCS, we do not expect any decline (i.e. inhibition) in behavioral performance in the cognitive and emotional Counting Stroop task as previous studies have shown that cathodal stimulation does not always result in decreased behavioral performance.

## Method

### Study design and procedure

**Figure 1:**
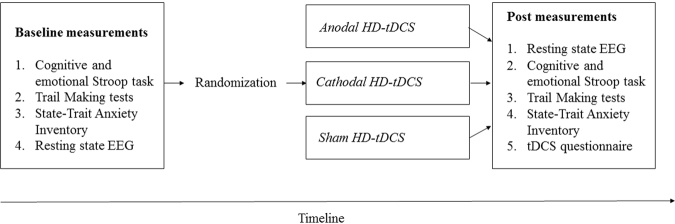
Study design.

The study was designed as a prospective, single-blinded, placebo controlled, randomized parallel-group study. Participants were first screened over the phone (e.g. handedness, HD-tDCS contraindications, neurological impairments) prior to enrolling into the study. Furthermore, study instructions were emailed to the participants to make sure that they abstain from alcohol for 24 hours prior to the study session, that they do not use any hair products (hair gel, hair spray, hair conditioner, etc.) on the day of the study session and that they do not consume any caffeinated products or nicotine for at least 1 hour prior to the study session. On the day of the study session, written informed consent was obtained from all individuals included in the study. The study was in accordance with the ethical standards of the Helsinki declaration (1964) and was approved by the Institutional Review Board of the University of Texas at Dallas. After baseline measurements (i.e. cognitive and emotional Counting Stroop task, 15 minutes; Trail Making tests, ~3 minutes; State-Trait Anxiety Inventory, ~2 minutes; resting-state EEG, ~30 minutes set-up, 5 minutes recording, ~15 minutes clean up), the patients were randomly assigned to one of the three groups: anodal HD-tDCS, cathodal HD-tDCS, or sham HD-tDCS targeting the dACC. All groups received one HD-tDCS procedure (~5 minutes set up, 20 minutes HD-tDCS, ~5 minutes clean up) followed by post measurements (i.e. resting-state EEG, ~30 minutes set-up, 5 minutes recording; cognitive and emotional Counting Stroop task, 15 minutes; Trail Making tests, ~3 minutes; State-Trait Anxiety Inventory, ~2 minutes) and a questionnaire about their HD-tDCS experience (approximately 5 minutes). The questionnaire was based on the tDCS adverse effects questionnaire proposed by Brunoni and colleagues (2011)^52^. The participants were compensated for their time completing the session. See Fig. 1 for study design.

### Participants

The participants were 45 healthy, right-handed adults (23 male, 22 female; mean age 23.52 years, *Sd* = 4.01 years). Their handedness was assessed by the Edinburgh Handedness Inventory^53^. A brief screening assessment determined that no one had a history of medical, neurological, or psychiatric disorders or any (HD-) tDCS contraindications. All subjects had normal or corrected to normal vision, no subject was taking medication or drugs and only 4 nicotine smokers were included in the study.

### High Definition–transcranial Direct Current Stimulation

**Figure 2:**
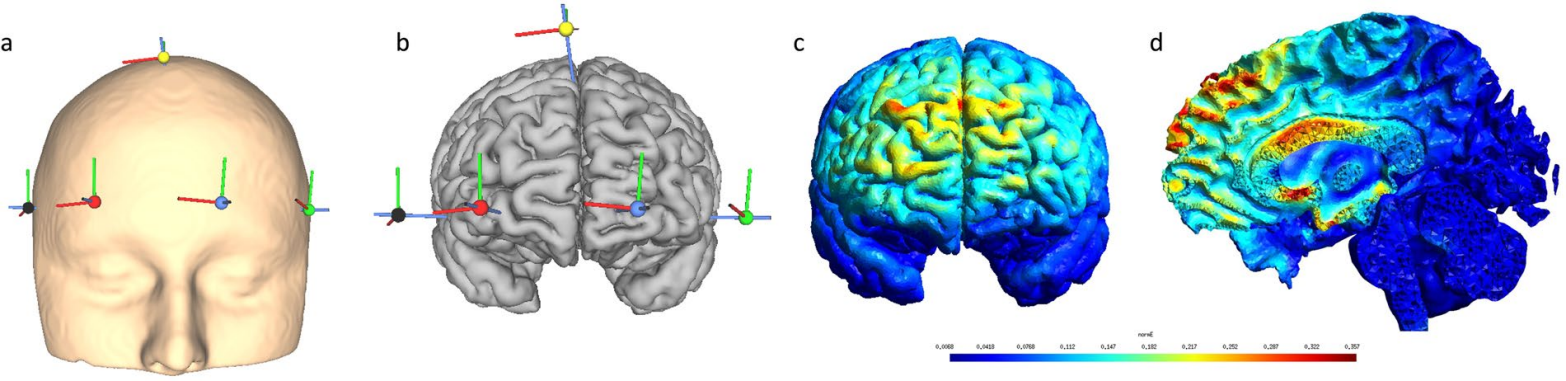
SimNIBS simulation of HD-tDCS study set up (**a**) head model setup, (**b**) brain model setup, (**c**) coronal view of current, (**b**) sagittal view of current.

Direct current was transmitted through 5 circular Ag/AgCl Electrodes (1 cm radius) with conductive gel on a neoprene head cap and delivered by a battery-driven, wireless multichannel transcranial current stimulator (Starstim tCS®, http://www.neuroelectrics.com). For all subjects, we used a montage where the central electrode was placed over the dACC and the 4 return electrodes were spread over the forehead. Having 1 target electrode and 4 return electrodes spread over the forehead have been chosen over a classic 4 × 1 ring HD-tDCS configuration (e.g. refs^36,41,44,45,54–56^) or a 2 × 2 HD-tDCS montage (e.g. ref.^36^) for several reasons. First, Dasilva and colleagues have found that these HD-tDCS montages (i.e. 4 × 1 ring configuration and 2 × 2 montage) enhanced the focality, but stimulated less deeply than the conventional tDCS montages^36^. Therefore, we opted to echo the conventional tDCS set up aiming to target a deeper brain region having one target electrode, but instead of having one large return electrode to reduce unwanted excitability changes under the return electrode^57,58^ we have opted to use multiple smaller return electrodes that were placed at a certain distance from the target electrode^59^. Furthermore, the choice of using a 5 electrode HD-tDCS configuration instead of conventional tDCS has the advantage that the 4 × 1 ring configuration has shown that the diffusion of return current along the four electrodes resulted in a more unidirectional modulation such that the polarity of the center electrode (anode or cathode) determines the primary change in excitation as opposed to the conventional tDCS where both anodal and cathodal effects must be considered^40,44,55,60^. Second, we intended to realize the peak electrical field somewhere midway between the center and return electrodes where the dACC is located, resembling a conventional tDCS configuration (except using 4 return electrodes instead of one big electrode)^37^. Previous research has demonstrated that the peak of the electrical field using the 4 × 1 HD-tDCS is right under the center electrode, whereas using conventional tDCS the electrical field peaked midway between the two electrodes instead of underneath one of them^37,61^. Using this configuration we aimed to control the direction of the electrical field going from the center electrode passing through the dACC, with the peak electrical field at the dACC, and then exiting at the forehead. We have used a computational model by ‘Simulation of Non-Invasive Brain Stimulation’ (SimNIBS, http://simnibs.de/) to cross validate our set-up showing that we would be able to stimulate the dACC before the start of the study (see Fig. 2). The site for stimulation was determined by the International 10/20 Electroencephalogram System corresponding to FZ for the central electrode and Fp1, Fp2, F7, and F8 for the return electrodes. For anodal stimulation (15 participants), the central electrode was programmed as the anode and for cathodal stimulation (15 participants) the central electrode was set as the cathode. The direct current was initially increased in a ramp-like fashion over 60 seconds until it reached 1 mA. The HD-tDCS stimulation procedure was maintained for a total of 20 minutes and then decreased in a ramp-like fashion over 60 seconds. Fifteen participants received sham HD-tDCS, in which the placement of the electrodes was identical to real HD-tDCS stimulation. The direct current in the sham procedure was also increased in a ramp-like fashion over 60 seconds until it reached 1 mA. Then, the current intensity was gradually reduced (ramp down) over 60 seconds until being switched off. This was followed by 20 minutes without active stimulation. Thus, the sham session lasted as long as the real HD-tDCS session to appropriately blind the procedure. The rationale behind this sham procedure was to mimic the transient skin sensation at the beginning of real HD-tDCS without producing any conditioning effects on the brain.

### Cognitive and emotional Counting Stroop Task

The cognitive and emotional Counting Stroop task is a combination of the Counting Stroop task and the emotional Counting Stroop task and was used in this study to explore the cognitive and emotional processing of the participants right before and right after the HD-tDCS procedure. The Counting Stroop task requires subjects to count the number of words in a display of words that denote a number (e.g. the word ‘three’ written twice) compared to a neutral condition (e.g. the word ‘dog’ written once) by pushing one of four buttons^14,48^. Reaction time to count these words is typically greater in the incongruent condition (e.g. the word ‘three’ written twice) compared to the neutral condition, i.e. the Stroop effect^14,48^. The emotional Counting Stroop task requires subjects to count the number of emotionally aroused words in a display of words that denote an emotion (e.g. the word ‘danger’ written twice) compared to a neutral condition (e.g. the word ‘cabinet’ written once) by pushing one of four buttons. It has been demonstrated in healthy subjects that highly aroused words elicit more interference than stimuli with low arousal^62^. Thus, the reaction time to count these emotionally aroused words is typically slower than counting neutral words (i.e. emotional interference).

In the cognitive blocks, the cognitive words represented written numbers (i.e. one, two, three, four) and the control words depicted animals (i.e. dog, cat, bird, fish), similar to the study of Bush and colleagues (1998). In the emotional blocks, the emotionally aroused words consisted of general negative words and the control words depicted household items, similar to the 1998 study by Whalen and Colleagues (see Table 1). The experiment consisted of six test blocks with a break after the third block. Block 1 and 2 were cognitive blocks that each consisted of 40 trials (12 consistent trials, 12 inconsistent trials, and 16 neutral trials randomly alternated), whereas Block 3 was an emotional block with 32 trials (16 emotional trials and 16 neutral trials randomly alternated). These three blocks were repeated after a 1 minute break where participants could take a break and relax their fingers. Each trial was displayed for 1.5 seconds followed by a fixation cross for 0.5 seconds (inter stimulus interval = 0.5 seconds). Prior to the six test blocks, the subjects first completed one practice block with five novel neutral words (i.e. porch, corridor, dishwasher, fan, mailbox) presented twice with feedback to make sure they practiced well enough on the task components of counting and button pressing, but without seeing the words to be tested in the test blocks. The experiment was displayed on a computer screen using Presentation software, and participants reported their answers by pushing one of four buttons on the keyboard using their right or left index or middle finger (1 = middle finger left hand, 2 = index finger left hand 3 = index finger right hand, 4 = middle finger right hand).

**Table 1:** Word stimuli used for present cognitive and emotional Counting Stroop Task.

Neutral (Cognitive)		Cognitive
Dog		One
Cat		Two
Bird		Three
Fish		Four
**Neutral (Emotional)**		**Emotional**
Table	Mirror	Violence
Closet	Bowl	Deceit
Corridor	Cabinet	Murder
Fan	Dishwasher	Contempt
Cushion	Mailbox	Painful
Curtain		Hazard
Pan		Torture
Porch		Danger

### Control assessments

#### State-Trait Anxiety Inventory (STAI)

This questionnaire was used to control for the possible^63^ influence of anxiety on the performance of the emotional Counting Stroop task^62^ and to measure possible effects of the HD-tDCS procedure on anxiety. The STAI consists of 40 statements, 20 measuring ‘trait’ anxiety and 20 measuring ‘state’ anxiety. In this study, ‘trait’ and ‘state’ anxiety were measured at baseline and only ‘state’ anxiety was measured after the HD-tDCS procedure. The ‘trait’ anxiety scale requires the participants to describe how they feel ‘in general’ on 20 statements, whereas the ‘state’ anxiety scale requires the participants to describe how they feel ‘now, at the present moment’ on 20 statements on a scale from 1 to 4 (1 = absolutely not, 2 = a little, 3 = much, 4 = very much). The higher the corrected total score, the greater the anxiety level.

#### Trail Making Tests (TMT) A and B

These tests were included to measure possible effects^64^ of the HD-tDCS procedure on general attention and executive function of alternating sequencing. TMT A measures processing speed and simple attention, while TMT B utilizes the same components as TMT A, plus the additional executive function of alternating sequencing. This test requires participants to connect a sequence of 25 consecutive targets on a sheet of paper as quickly as possible. For TMT A, the targets are all numbers and the task is to connect the numbers in sequential order (i.e. 1, 2, 3, …), while for TMT B, the targets alternate between numbers and letters (i.e. 1, A, 2, B, …).

### Resting-state Electroencephalography and source localization

Electroencephalography (EEG) data collection recordings (Neuroscan, http://compumedicsneuroscan.com/) were obtained in a quiet room while each participant was sitting upright in a comfortable chair. The participants’ EEGs were collected immediately before and after HD-tDCS. The scalp was cleaned with alcohol wipes before the baseline EEG recording. After the baseline EEG recoding, the hair was washed and dried before the HD-tDCS session. After the HD-tDCS session, the conductive gel was cleaned up before the post EEG recoding. The EEG was sampled with 64 electrodes in the standard 10–10 International placement and impedances were checked to remain below 5 kΩ. Data were collected eyes-closed (sampling rate = 1 kHz, band passed DC–200 Hz) and collection took ~ 5 minutes. The midline reference was located at the vertex and the ground electrode was located at AFZ. Participants were instructed not to drink alcohol for 24 hours prior to EEG recording or caffeinated beverages one hour before recording to avoid alcohol- or caffeine-induced changes in the EEG stream^65–67^. The alertness of participants was checked by monitoring both slowing of the alpha rhythm and appearance of spindles in the EEG stream to prevent possible enhancement of the theta power due to drowsiness during recording^68^. No participants included in the current study showed such EEG changes during measurements. The data were then resampled to 128 Hz, band-pass filtered (fast Fourier transform filter) to 2–44 Hz, and re-referenced to the average reference using EEGLAB 13_1_1b. The data were then plotted in EEGLAB for a careful inspection of artifacts and all episodic artifacts suggestive of eye blinks, eye movements, jaw tension, teeth clenching, or body movement were manually removed from the EEG stream.

Standardized low-resolution brain electromagnetic tomography (sLORETA) was used to estimate the intracerebral electrical sources that generated the scalp-recorded activity in each of the 8 frequency bands, i.e., delta (2–3.5 Hz), theta (4–7.5 Hz), alpha1 (8–10 Hz), alpha2 (10.5–12 Hz), beta1 (12.5–18 Hz), beta2 (18.5–21 Hz), beta3 (21.5–30 Hz), and gamma (30.5–44 Hz). Standardized LORETA computes electrical neuronal activity as current density (A/m2) without assuming a predefined number of active sources. The sLORETA solution space consists of 6239 voxels (voxel size 5 × 5 × 5 mm) and is restricted to cortical gray matter and hippocampi, as defined by the digitized Montreal Neurological Institute (MNI) 152 template^69^. Scalp electrode coordinates on the MNI brain are derived from the international 10–20 system^70^.

The Tomography sLORETA has received validation from studies combining LORETA with other more established methods such as fMRI^71,72^, structural MRI^73^ and PET^74–76^. Further sLORETA validation has been based on accepting as ground truth that the localization findings obtained from invasive implanted depth electrodes, in which case there are several studies in epilepsy^77,78^ and cognitive ERPs^79^. It is worth mentioning that deep structures such as the anterior cingulate cortex^80^, and medial temporal lobes^81^ can be correctly localized with these methods. In the current implementation of sLORETA, computations were made in a realistic head model^69^, using the MNI 152 template^82^, with the three-dimensional solution space restricted to cortical gray matter, as determined by the probabilistic Talairach atlas^83^. The standard electrode positions on the MNI 152 scalp were taken from^70^ and^84^. The intracerebral volume in partitioned in 6239 voxels at 5 mm spatial resolution. Thus, sLORETA images represent the standardized electrical activity at each voxel in neuroanatomic Montreal Neurological Institute (MNI) space as the exact magnitude of the estimated current density. Anatomical labels as Brodmann areas are also reported using MNI space, with correction to Talairach space^85^.

### Data Analysis

#### Behavioral data

SPSS version 22.0 was used for all statistical analyses on the behavioral data. For the cognitive Counting Stroop task, we applied a repeated measures ANOVA with stimulation (anodal HD-tDCS, cathodal HD-tDCS, sham HD-tDCS) as the between-subjects variable and time (pre vs. post stimulation) and condition (congruent vs. incongruent) as within-subjects variables. A similar analysis was applied for the emotional Counting Stroop task. These analysis were applied on the entire sample as well as on a sample where the 4 nicotine smokers in our sample (1 in anodal, two in cathodal and 1 in sham group) were excluded to control for any effect of nicotine withdrawal on Stroop task performance^86–88^. For the STAI and the Trail Making tests a repeated measures ANOVA was applied with stimulation (anodal HD-tDCS, cathodal HD-tDCS and sham HD-tDCS) as the between subjects variable, and time point (pre vs post stimulation) for the STAI and the Trail Making tests, respectively, as within subjects variable. To further analyze the conditional effects, we used two-sample t-tests.

#### Resting-state EEG data

Average Fourier cross-spectral matrices were computed for bands delta (2–3.5 Hz), theta (4–7.5 Hz), alpha1 (8–10 Hz), alpha2 (10.5–12 Hz), beta1 (12.5–18 Hz), beta2 (18.5–21 Hz), beta3 (21.5–30 Hz), and gamma (30.5–44 Hz). Source localization Standardized low-resolution brain electromagnetic tomography (sLORETA) was used to estimate the intracerebral electrical sources that generated the scalp-recorded activity in each of the eight frequency bands^89^. The methodology used is a non-parametric permutation test. It is based on estimating, via randomization, the empirical probability distribution for the max-statistic under the null hypothesis comparisons^90^. This methodology corrects for multiple testing (i.e. for the collection of tests performed for all voxels, and for all frequency bands). Due to the non-parametric nature of this method, its validity does not rely on any assumption of Gaussianity^90^. The significance threshold for all tests was based on a permutation test with 5000 permutations. Comparisons were made between the pre and post HD-tDCS measurements. The comparison was performed on a whole brain by sLORETA statistical contrast maps through multiple voxel-by-voxel comparisons in a logarithm of *t*-ratio.

## Results

### Demographics

A comparison between the three groups (anodal, cathodal and sham) revealed no significant effect between the different groups for age (*F* = 0.51, *p* = 0.61) and gender (χ^2^ = 0.37, *p* = 0.93).

### High Definition–transcranial Direct Current Stimulation experience

HD-tDCS was well tolerated and no HD-tDCS related complications were noted by the participants or the experimenter during the HD-tDCS procedure. The most common side-effects related to the HD-tDCS procedure, as reported by active and sham HD-tDCS groups, were tingling (real: 33.33% and sham: 58.33%), itching (real: 33.33% and sham: 33.33%), and burning sensation (real: 22.22% and sham: 13.33%). In this study, 57% of the participants were able to guess what condition they received (real or sham tDCS) using this conventional sham method.

### Control variables

#### STAI

The mean trait anxiety score in our sample was 34.39 (SD = 8.84) and the mean state anxiety score was 29.09 (*Sd* = 8.72) at baseline. A univariate ANOVA revealed that there were no significant differences between the three groups (anodal-cathodal-sham) in the baseline measure on trait (*F* = 0.27, *p* = 0.76) or state (*F* = 0.43, *p* = 0.65) anxiety. To assess the effect of HD-tDCS on anxiety, we compared the mean state anxiety scores by stimulation group (anodal-cathodal-sham) over time (pre-post) using a repeated measures ANOVA. The repeated measures ANOVA did not show a significant main effect for time (pre-post) (*F* = 2.08, *p* = 0.16) nor did it show a significant interaction effect (time x group) (*F* = 1.46, *p* = 0.25).

#### Trail Making Tests A and B

To assess the effect of HD-tDCS on the performance of Trails A and B, we compared the average reaction time (in seconds) by stimulation group (anodal-cathodal-sham) over time (pre-post) using repeated measures ANOVA. Neither Trail A (*F* = 1.33, *p* = 0.28) nor Trial B (*F* = 1.28, *p* = 0.29) yielded a significant interaction effect between time x stimulation group, but there was a significant main effect for time for Trails A (*F* = 22.83, *p* < 0.001) and B (*F* = 19.21, *p* < 0.001). For all groups we found that post-stimulation performance (Anodal *M* = 18.17 s, *Sd* = 6.17; Cathodal *M* = 17.96 s, *Sd* = 4.56; Sham *M* = 18.32 s, *Sd* = 4.60) was faster than pre-stimulation performance (Anodal *M* = 22.93 s, *Sd* = 6.43; Cathodal *M* = 20.12 s, *Sd* = 3.60; Sham *M* = 23.86 s, *Sd* = 8.54) for Trail A. For Trail B, post-stimulation (Anodal *M* = 38.04 s, *Sd* = 7.06; Cathodal *M* = 36.67 s, *Sd* = 12.85; Sham *M* = 37.42 s, *Sd* = 9.71) also showed faster performance than pre-stimulation (Anodal *M* = 44.33 s, *Sd* = 6.90; Cathodal *M* = 39.66 s, *Sd* = 11.10; Sham *M* = 45.37 s, *Sd* = 15.84).

### The cognitive blocks of the Counting Stroop Task - Reaction times and errors

A comparison between the three different groups (anodal, cathodal and sham) at baseline showed no significant difference for the congruent (*F* = 0.64, *p* = 0.53) or the incongruent trials (*F* = 0.35, *p* = 0.71). In addition, we have compared the three different groups (anodal, cathodal and sham) after stimulation showing no significant difference for the congruent (*F* = 0.39, *p* = 0.68) as well as for the incongruent trials (*F* = 0.22, *p* = 0.80).

**Figure 3:**
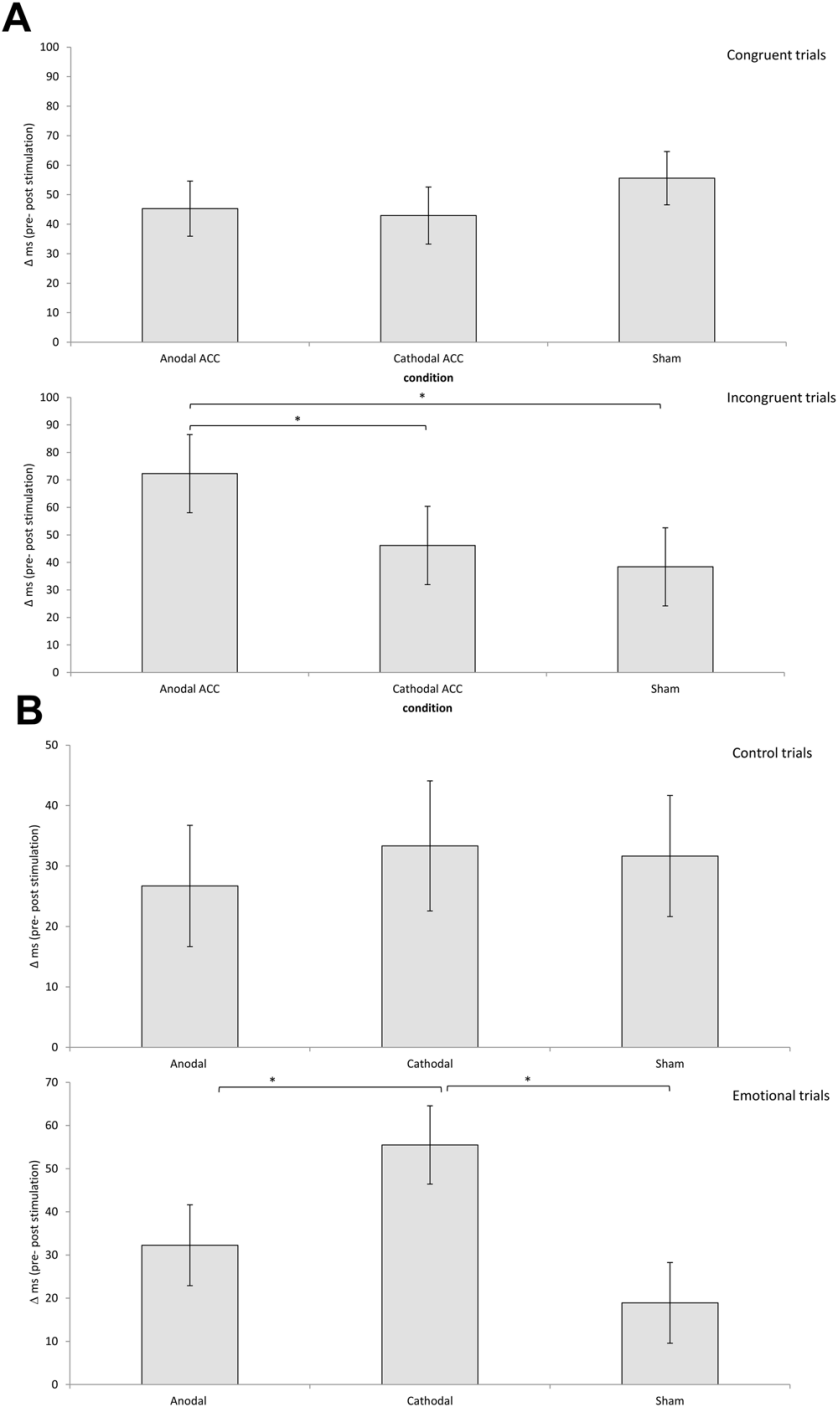
(**A**) A comparison between the effect of HD-tDCS (=pre–post) showed no significant difference between the three groups (anodal, cathodal, sham) for the congruent trials while for the incongruent trials the anodal group was faster after HD-tDCS targeting the dorsal anterior cingulate cortex in comparison to the cathodal or sham groups. (**B**) A comparison between the effect of HD-tDCS (=pre–post) showed no significant difference between the three groups (anodal, cathodal, sham) for the control trials, while on the emotional trials, the cathodal group was faster after HD-tDCS targeting the dorsal anterior cingulate cortex in comparison to the anodal or sham groups.

To assess the efficacy of HD-tDCS targeting the dACC, we compared the mean reaction time on the cognitive blocks of the Counting Stroop task by applying a repeated measures ANOVA with stimulation group (anodal-cathodal-sham) as the between-subjects variable and time (pre vs. post stimulation) and condition (congruent vs. incongruent) as within-subjects variables. The analysis revealed a significant main effect for condition (*F* = 153.37, *p* < 0.001) as well as for time (*F* = 60.40, *p* < 0.001). For condition, we saw that subjects in general were slower for incongruent trials (*M* = 624.63 ms, *Sd* = 86.50) in comparison to congruent trials (*M* = 543.66 ms, *Sd* = 57.74). For time, we found that post-stimulation (*M* = 559.26 ms, *Sd* = 57.74) responses in general were faster than pre-stimulation (*M* = 609.03 ms, *Sd* = 51.99). In addition, a significant three-way interaction effect was obtained between time × condition × group (*F* = 3.74, *p* = 0.033) (See Table 2). Further exploration of this effect showed that there was no significant difference between the three groups for the congruent trials (*t* = 0.18–0.88, *p* = 0.39–0.86), while for the incongruent trials the anodal group subjects responded faster after HD-tDCS targeting the dACC in comparison to the cathodal group subjects after cathodal HD-tDCS targeting the dACC (*t* = 2.14, *p* = 0.041) or the sham group subjects after the sham procedure (*t* = 2.26, *p* = 0.032) (see Fig. 3). The significant three-way interaction between time × condition × group remained even when excluding the 4 smokers (1 in anodal, 2 in cathodal and 1 in sham group) from the analysis (*F* = 4.22, *p* = 0.023). To investigate the effect of anxiety on the counting Stroop performance, we added state anxiety as a covariate to the analysis. Adding state anxiety as covariate to the analysis did not affect the three-way interaction (*F* = 0.45, *p* = 0.51) and the significant effect remained. No significant difference was demonstrated between the cathodal and the sham group (*t* = 0.38, *p* = 0.71) (see Fig. 3). No significant effect was shown for group (*F* = 0.08, *p* = 0.92), or for the two-way interaction of condition × group (*F* = 0.54, *p* = 0.59), time × group (*F* = 0.40, *p* = 0.67), or condition × time (*F* = 0.33, *p* = 0.57).

**Table 2:** Reaction times for pre and post anodal, cathodal, sham HD- tDCS targeting the dorsal anterior cingulate cortex for the cognitive and emotional Stroop task.

	Group	Stimulation		Difference
		Pre (ms) (SD)	Post (ms) (SD)	(ms) (SD)
Congruent Trials	Anodal	568.64 (65.01)	523.38 (53.18)	45.26 (34.92)
	Cathodal	563.42 (62.81)	520.48 (51.38)	42.94 (36.24)
	Sham	570.82 (67.47)	515.24 (55.19)	55.58 (33.73)
Incongruent Trials	Anodal	663.29 (93.68)	591.01 (82.81)	72.28 (53.08)
	Cathodal	637.13 (90.50)	590.98 (79.65)	46.15 (53.28)
	Sham	650.88 (92.21)	612.49 (85.55)	38.39 (53.08)
Emotional Trials	Anodal	590.287 (63.82)	558.040 (63.70)	32.25 (35.03)
	Cathodal	584.577 (68.56)	529.085 (68.40)	55.49 (33.88)
	Sham	564.167 (63.82)	545.253 (63.70)	18.91 (35.03)
Control Trials	Anodal	584.737 (60.81)	558.027 (63.29)	26.71 (37.48)
	Cathodal	574.950 (65.33)	541.623 (67.98)	33.33 (40.26)
	Sham	571.923 (60.81)	540.267 (63.33)	31.66 (37.48)

A repeated measures ANOVA on the number of errors with stimulation group (anodal-cathodal-sham) as the between-subjects variable and time (pre- vs. post stimulation) and condition (congruent vs. incongruent) as within-subjects variables revealed no significant effects.

### The emotional blocks of the Counting Stroop Task - Reaction times and errors

A comparison between the three different groups (anodal, cathodal and sham) at baseline showed no significant difference for the emotional (*F* = 0.69, *p* = 0.51) as well as for the neutral trials (*F* = 0.58, *p* = 0.57). In addition, we compared the three different groups (anodal, cathodal and sham) after stimulation showing no significant difference for the emotional (*F* = 0.31, *p* = 0.74) as well as for the neutral trials (*F* = 0.34, *p* = 0.72).

A repeated measures ANOVA with stimulation group (anodal-cathodal-sham) as the between- subjects variable and time (pre- vs. post- stimulation) and condition (emotional vs. control) as the within-subjects variables was conducted. This analysis revealed a main effect for time (*F* = 33.98, *p* < 0.001), indicating that post-stimulation (*M* = 547.05, *Sd* = 64.01) responses in general were faster than pre-stimulation (*M* = 578.44, *Sd* = 62.05). Also, a significant three-way interaction was obtained between time × condition × group (*F* = 3.45, *p* = 0.042) (See Table 2). Further exploration of this effect showed that there was no significant difference between the three groups for the control trials (*t* = 0.11–0.46, *p* = 0.65–0.91). For the emotional trials, the cathodal group subjects were found to respond faster after HD-tDCS targeting the dACC in comparison to the anodal group subjects after anodal HD-tDCS targeting the dACC (*t* = 1.74, *p* = 0.047) or the sham group subjects after the sham procedure (*t* = 1.98, *p* = 0.028) (see Fig. 3). The significant three-way interaction between time × condition × group remained even when excluding the 4 smokers (1 in anodal, 2 in cathodal and 1 in sham group) from the analysis (*F* = 3.16, *p* = 0.049). To investigate the effect of anxiety on emotional processing, i.e. the emotional counting Stroop performance, we added state anxiety as a covariate to the analysis. Adding state anxiety (*F* = 0.09, *p* = 0.76) as a covariate to the analysis did not affect the three-way interaction, the significant effect remained. No significant main effects were obtained for condition (*F* = 0.35, *p* = 0.56), or group (*F* = 0.09, *p* = 0.76), and no significant two-way interactions for condition × group (*F* = 0.40, *p* = 0.67), time × group (*F* = 0.35, *p* = 0.70), and condition × time (*F* = 0.16, *p* = 0.69) were found.

A repeated measures ANOVA on the number of errors with stimulation group (anodal-cathodal-sham) as the between-subjects variable and time (pre- vs. post-stimulation) and condition (congruent vs incongruent) as within-subjects variables revealed no significant effects.

### Resting-state EEG

A comparison between the 3 different groups (anodal, cathodal and sham) at baseline (before stimulation) did not show any significant effects when applying a pairwise comparison for the delta, theta, alpha1, alpha2, beta1, beta2, beta3, and gamma frequency bands.

**Figure 4:**
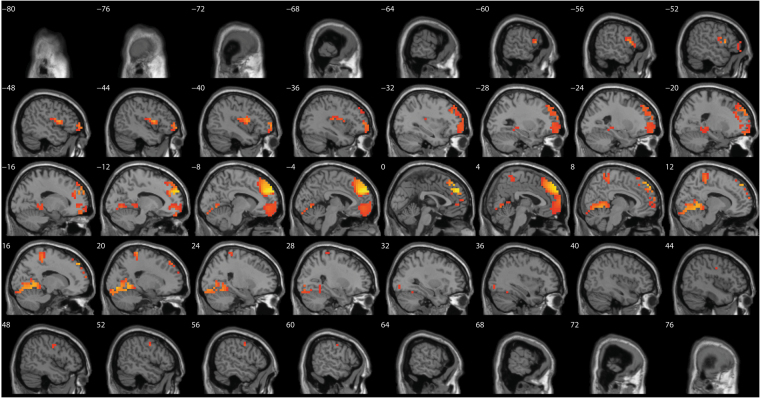
A comparison between before and after source localized EEG for the anodal group revealed a significant difference for the beta3 frequency band (*F* = 4.82, *p* < 0.01) showing increased activity overlaying the anterior cingulate cortex, pre-supplementary motor cortex extending into the orbitofrontal cortex after anodal stimulation in comparison to pre-stimulation. No significant effects were obtained for the delta, theta, alpha1, alpha2, beta1, beta2, and gamma frequency bands.

A comparison between before and after source-localized EEG for the anodal group only revealed a significant difference for the beta3 frequency band (*F* = 4.82, *p* < 0.01) (see Fig. 4). This analysis showed increased activity overlaying the anterior cingulate cortex, pre-supplementary motor cortex extending into the orbitofrontal cortex after anodal stimulation in comparison to pre-stimulation. No significant effect was obtained for the delta, theta, alpha1, alpha2, beta1, beta2, and gamma frequency bands when comparing before and after whole brain source-localized EEG recording.

**Figure 5:**
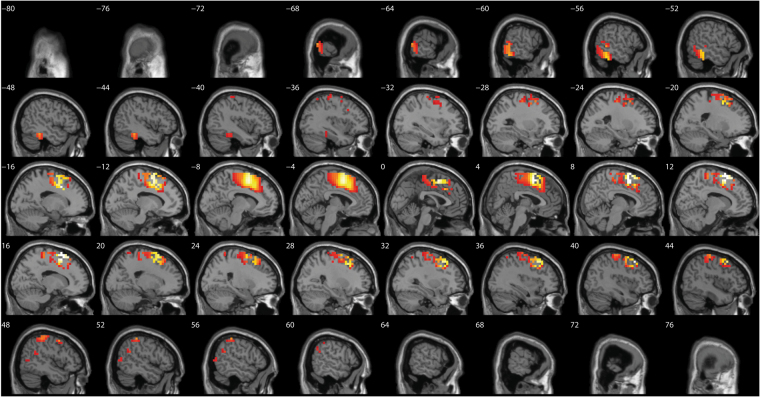
A comparison between before and after source-localized EEG for the cathodal group showed a significant difference for the theta frequency band (*F* = 3.63, *p* < 0.05) showing increased activity overlaying the dorsal anterior cingulate cortex extending to the rostral anterior cingulate cortex after cathodal stimulation in comparison to pre-stimulation. No significant effects were obtained for the delta, alpha1, alpha2, beta1, beta2, beta3 and gamma frequency bands.

A comparison between before and after source-localized EEG for the cathodal group only showed a significant difference for the theta frequency band (*F* = 3.63, *p* < 0.05) (see Fig. 5). This analysis showed increased activity overlaying the dACC extending into the rostral anterior cingulate cortex after cathodal stimulation in comparison to pre-stimulation. No significant effect was obtained for the delta, alpha1, alpha2, beta1, beta2, beta3, and gamma frequency band throughout the whole brain.

A comparison between before and after source-localized EEG for the sham group demonstrated no significant effect for the delta, theta, alpha1, alpha2, beta1, beta2, beta3, and gamma frequency bands throughout the whole brain.

**Figure 6:**
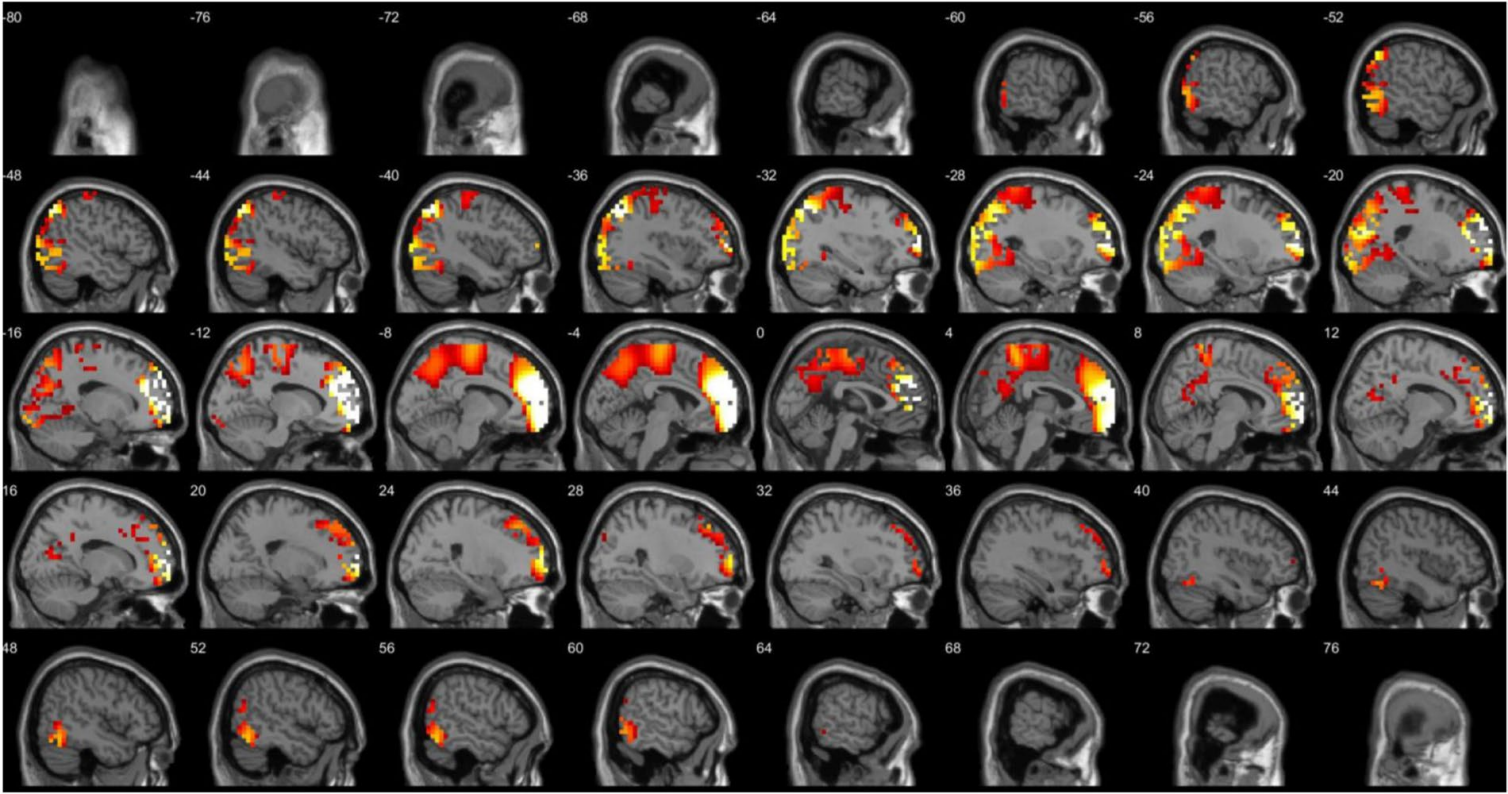
A comparison between the three different groups (anodal, cathodal and sham) after stimulation revealed different significant effects. The comparison between anodal and sham stimulation demonstrated a significant difference for the beta3 frequency band (*F* = 2.33, *p* < 0.01). This analysis showed increased activity overlaying the dACC extending into the pregenual anterior cingulate cortex, the posterior cingulate cortex, and orbitofrontal cortex, for anodal stimulation in comparison to sham stimulation. No significant effect was obtained for the delta, theta, alpha1, alpha2, beta1, beta2 and gamma frequency bands.

**Figure 7:**
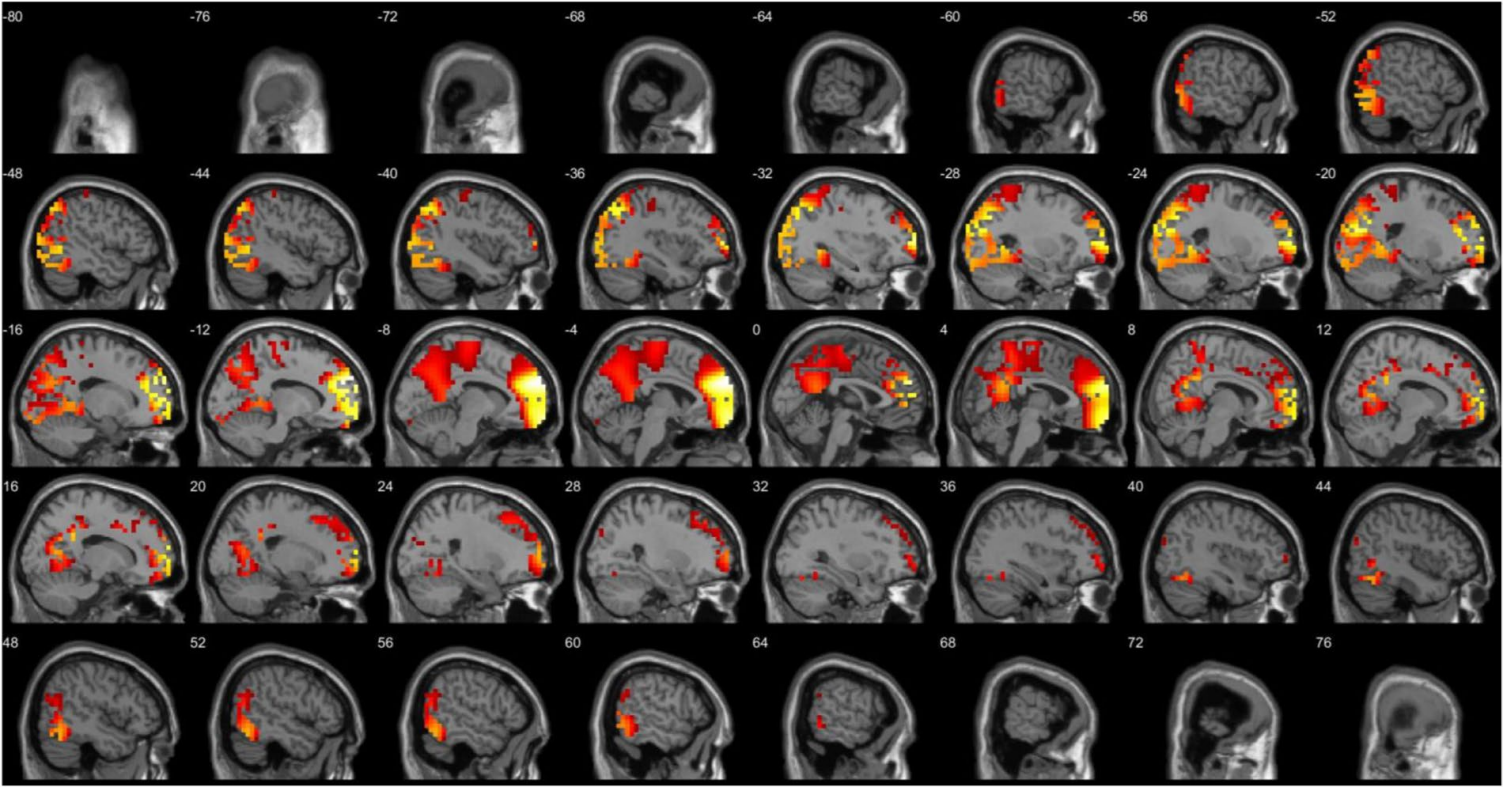
A comparison between the three different groups (anodal, cathodal and sham) after stimulation revealed different significant effects. The comparison between cathodal and sham stimulation showed a significant difference for the theta frequency band (*F* = 2.83, *p* < 0.05) (see Fig. 7). This analysis showed increased activity overlaying the rostral anterior cingulate cortex extending into the pregenual anterior cingulate cortex, ventromedial prefrontal cortex, and orbitofrontal cortex and to a smaller extend to the posterior cingulate cortex after cathodal stimulation in comparison to sham stimulation. No significant effect was obtained for the delta, alpha1, alpha2, beta1, beta2, beta3, and gamma frequency band.

**Figure 8:**
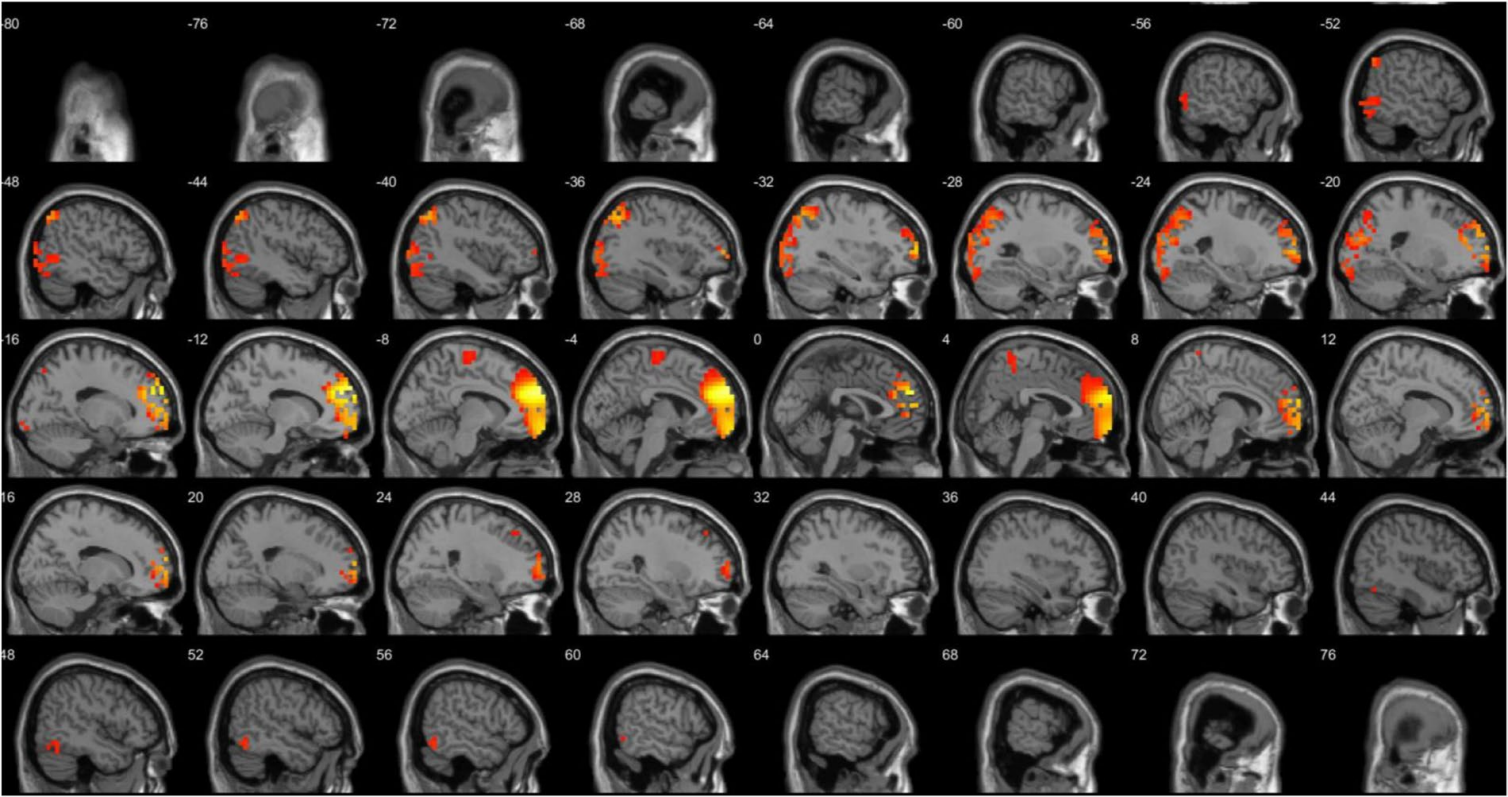
A comparison between the three different groups (anodal, cathodal and sham) after stimulation revealed different significant effects. For the comparison between anodal and cathodal stimulation it showed a significant difference for the beta3 frequency band (*F* = 2.64, *p* < 0.05) (see Fig. 8). This analysis showed increased activity overlaying the anterior cingulate cortex extending into the pregenual anterior cingulate cortex, ventromedial prefrontal cortex for anodal stimulation in comparison to cathodal stimulation. No significant effect was obtained for the delta, theta, alpha1, alpha2, beta1, beta2, and gamma frequency bands.

A comparison between the three different groups (anodal, cathodal and sham) after stimulation revealed different significant effects. The comparison between anodal and sham stimulation demonstrated a significant difference for the beta3 frequency band (*F* = 2.33, *p* < 0.01) (see Fig. 6). This analysis showed increased activity overlaying the dACC extending into the pregenual anterior cingulate cortex, the posterior cingulate cortex, and orbitofrontal cortex, for anodal stimulation in comparison to sham stimulation. No significant effect was obtained for the delta, theta, alpha1, alpha2, beta1, beta2 and gamma frequency bands. The comparison between cathodal and sham stimulation showed a significant difference for the theta frequency band (*F* = 2.83, *p* < 0.05) (see Fig. 7). This analysis showed increased activity overlaying the rostral anterior cingulate cortex extending into the pregenual anterior cingulate cortex, ventromedial prefrontal cortex, and orbitofrontal cortex and to a smaller extend to the posterior cingulate cortex after cathodal stimulation in comparison to sham stimulation. No significant effect was obtained for the delta, alpha1, alpha2, beta1, beta2, beta3, and gamma frequency band. For the comparison between anodal and cathodal stimulation it showed a significant difference for the beta3 frequency band (*F* = 2.64, *p* < 0.05) (see Fig. 8). This analysis showed increased activity overlaying the anterior cingulate cortex extending into the pregenual anterior cingulate cortex, ventromedial prefrontal cortex for anodal stimulation in comparison to cathodal stimulation. No significant effect was obtained for the delta, theta, alpha1, alpha2, beta1, beta2, and gamma frequency bands.

## Discussion

In this study, we investigated whether anodal and cathodal HD-tDCS were capable of modulating dorsal anterior cingulate cortex (dACC) activity, reflected in neurophysiological and behavioral changes. Source-localized resting-state EEG before and after HD-tDCS showed significant changes after both anodal and cathodal stimulation. Behavioral changes were also found after anodal and cathodal HD-tDCS. In this section, we will discuss our behavioral and neurophysiological findings in light of previous research findings and theories.

As expected, our results show a general Stroop effect in the cognitive Counting Stroop task at baseline. Participants showed significantly slower performance on the incongruent trials when compared to the congruent trials, which falls in line with previous literature^14,48^. For the emotional Counting Stroop task, we did not demonstrate an emotional interference effect in the emotional trials compared to the control trials at baseline, which is consistent with the findings of Whalen and colleagues^49^. It has been suggested that the degree that this version of the emotional Counting Stroop task represents a true ‘Stroop’ interference task, in the sense that emotional words will increase motor-response times compared to neutral words, depends upon the subjects of the study and the words that are presented^91^. Much research on the emotional Stroop task has demonstrated that interference effects are observed in psychopathological groups in response to words that are specific to their disorder, and in normal subjects when the words are related to current concerns endorsed by them^91^. It may be possible that the emotional words (i.e. general negative words from Whalen *et al*., 1998) in the task did not tap into the current concerns of our sample of healthy subjects resulting in the absence of an emotional interference effect at baseline.

Significant behavioral changes were also demonstrated after anodal and cathodal HD-tDCS targeting the dACC as measured by the cognitive and emotional Counting Stroop task. Our results show that participants responded significantly faster after the HD-tDCS procedure (anodal, cathodal, sham) when compared to their performance before the procedure in both the cognitive and the emotional parts of the Counting Stroop task. This might be due to a practice effect, but a closer look at the data shows that anodal and cathodal HD-tDCS generated differential performance effects. More specifically, the anodal HD-tDCS group was only faster in the incongruent trial (and not in the congruent trial) when compared with the cathodal and sham groups. On the other hand, the cathodal HD-tDCS group responded significantly faster in the emotional trials (and not in the neutral trials) when compared with the anodal and sham groups post stimulation. Previous research has suggested a more general function for the dACC as this brain region has been demonstrated to be involved in cognitive, affective, sensory and autonomic processing^92–94^ and also since the anterior cingulate cortex has deliberately been targeted in lesioning techniques (i.e. cingulotomy psychosurgery) and in neuromodulation techniques (e.g. deep brain stimulation, TMS) for the treatment of various disorders. However, using anodal HD-tDCS in our study only improved the performance on the cognitive component of the Counting Stroop task, not the emotional component.

Contradictory to our hypothesis, cathodal HD-tDCS targeting the dACC did reveal a facilitatory effect in the emotional trials (and not the neutral trials) of the emotional Counting Stroop task, even though there was no emotional interference effect found at baseline and even when controlling for trait or state anxiety. These findings do not support the conventional “anodal-excitation and cathodal-inhibition dual polarity hypothesis”, as cathodal HD-tDCS did not result in slower performance on the emotional and cognitive part of the Counting Stroop task^33–35^. Previous research has already indicated that the relationship between facilitation and inhibition is often quite complex^33–35^. The anodal-excitation and cathodal-inhibition dual polarity effect, being anodal tDCS facilitating and cathodal tDCS worsening behavioral outcome^32,33^, has not always been observed in all tDCS studies^34^. The effect occurs quite commonly in motor investigations, but only rarely in cognitive studies^34^. The facilitatory cathodal effect in the emotional Counting Stroop task cannot be explained by this theory.

As expected, the changes in cognitive and emotional processing due to anodal and cathodal HD-tDCS targeting the dACC cannot be explained by general attention or executive function as measured by Trail Making Tests A and B. However, these changes can partially be contributed by pre-supplementary motor area (pre-SMA) activity changes as targeting the dACC with our HD-tDCS set up inevitably passes through the pre-SMA area. The pre-SMA has been associated with inhibitory control in healthy subjects^95,96^ as well as in patient populations, including obsessive-compulsive disorder^97,98^. In healthy subjects, anodal transcranial direct current stimulation targeting the pre-SMA improved efficiency of inhibitory control^96^ in a stop-signal task, whereas cathodal tDCS showed a tendency towards impaired inhibitory control^95^. Similar results were found using rTMS targeting the pre-SMA^99,100^. Therefore, findings of any cognitive and emotional processing changes due to our HD-tDCS set up have to be interpreted with caution.

Significant neurophysiological changes, measured with source-localized resting-state EEG, were also observed after anodal and cathodal HD-tDCS targeting the dACC. After stimulation, subjects in the anodal HD-tDCS condition showed a significant increase in beta frequency band activity in the dACC, while cathodal HD-tDCS led to a significant increase in activity at the dorsal and rostral anterior cingulate cortex in the theta frequency band. These neurophysiological findings fit with the behavioral findings indicating that cathodal HD-tDCS targeting the dACC does not follow the “anodal-excitation and cathodal-inhibition dual polarity hypothesis”. However, any connection between the neurophysiological and behavioral results in our study needs to be interpreted with caution, as the EEG data were not recorded during task performance. Interestingly, the altered activity after cathodal HD-tDCS in this study was also seen in a more rostral anterior cingulate cortex area, whereas the altered activity after anodal HD-tDCS was only found in the targeted dACC area. This might suggest that different groups of neurons might react to the different types of stimulation (anodal vs. cathodal). Davis and colleagues (2005) have investigated neuronal activity during the cognitive and emotional Counting Stroop task and have demonstrated that the dACC contains a large group of neurons that respond to conflict interference in general, acting as salience detectors when faced with conflict or emotional stimuli. They further found that the dACC also contained a small group of cells that was responsive only in the emotional Counting Stroop task, but not in the cognitive Counting Stroop task^101^. They suggest that the impact of this last type of neurons on behavior and cognition may depend on the balance of activity contributed by this neuron type within the dorsal and rostral anterior cingulate cortex^101^. Based on these findings^101^, we can hypothesize that the group of neurons involved in general (cognitive and emotional) interference may have reacted to both anodal and cathodal HD-tDCS, whereas the group of neurons only responsive to emotional trials may have reacted solely to cathodal HD-tDCS when we were targeting the dACC. This hypothesis fits with our imaging findings as anodal HD-tDCS only demonstrated changes in the dACC and cathodal HD-tDCS indicated changes in both dorsal and rostral anterior cingulate cortex.

Interestingly, previous research has already associated theta/beta ratio and theta/beta coupling with attentional control, response inhibition, and both affective traits and performance on emotional-cognitive experimental tasks^102–104^. In the context of the cognitive Stroop task specifically, it has been shown that the beta frequency band is sensitive to discrimination between congruent and incongruent items and that increased coherence between the left frontal and left parietal areas was observed for incongruent trials compared to congruent trials^105^. This is consistent with a more recent time-frequency analysis study that shows greater association between the left frontal and parietal areas during incongruent tasks than in congruent tasks within a time interval of 100–400 ms in the beta frequency band. Changes in the beta frequency band have previously been found after 20 minutes of anodal tDCS targeting the prefrontal and motor cortices, suggesting that anodal tDCS changes the “ready state” to perform cognitive or motor tasks^106,107^. Taken together, these findings support our data, which show that anodal stimulation modulates the beta frequency band. It also is consistent with data that shows that incongruent trials show greater modulation than the congruent trials in the cognitive Counting Stroop task. On the other hand, the theta frequency band has been associated with post-response monitoring and is believed to reflect voluntary control processing^108–110^, emotional processing^111^, action regulation^112,113^, and emotional decision making^114^.Cathodal tDCS has been demonstrated to significantly increase both theta rhythms and local connectivity in the theta band range^115,116^. This pattern is also demonstrable in animals; increased theta activity in the cerebral cortex of cats has been demonstrated after cathodal tDCS^117^. It might be possible that participants at perform better on the emotional Counting Stroop task due to increasing theta activity after cathodal tDCS. It is however not clear why this is selective for the emotional trials and not for the control trials.

In conclusion, this study demonstrated that HD-tDCS is able to modulate activity in the dACC, suggesting that it has potential for use as a treatment tool. This finding may have implications for the development of new HD-tDCS treatment protocols for neuropsychiatric disorders and has the potential to replace invasive or other non-invasive neuromodulation techniques. The stimulation protocol is feasible for routine clinical treatment and was well tolerated by all participants. Future studies should investigate the effects of anodal and cathodal HD-tDCS in healthy subjects as well as in patient populations.

## References

[1] Gasquoine PG (2013). Localization of function in anterior cingulate cortex: from psychosurgery to functional neuroimaging. Neurosci. Biobehav. Rev..

[2] Downar J, Blumberger DM, Daskalakis ZJ (2016). The Neural Crossroads of Psychiatric Illness: An Emerging Target for Brain Stimulation. Trends Cogn. Sci..

[3] Goodkind M (2015). Identification of a common neurobiological substrate for mental illness. JAMA psychiatry.

[4] Harmer CJ, Thilo KV, Rothwell JC, Goodwin GM (2001). Transcranial magnetic stimulation of medial-frontal cortex impairs the processing of angry facial expressions. Nat. Neurosci..

[5] Jahanshahi M, Rothwell J (2000). Transcranial magnetic stimulation studies of cognition: an emerging field. Exp. Brain Res..

[6] Walsh V, Rushworth M (1999). A primer of magnetic stimulation as a tool for neuropsychology. Neuropsychologia.

[7] Russo JF, Sheth SA (2015). Deep brain stimulation of the dorsal anterior cingulate cortex for the treatment of chronic neuropathic pain. Neurosurg. Focus.

[8] Boccard SG (2014). Deep brain stimulation of the anterior cingulate cortex: targeting the affective component of chronic pain. Neuroreport.

[9] Boccard, S. G. *et al*. Targeting the affective component of chronic pain: a case series of deep brain stimulation of the anterior cingulate cortex. *Neurosurgery* **74**, 628–635; discussion 635–627, 10.1227/NEU.0000000000000321 (2014). 10.1227/NEU.0000000000000321 24739362

[10] Spooner J, Yu H, Kao C, Sillay K, Konrad P (2007). Neuromodulation of the cingulum for neuropathic pain after spinal cord injury. Case report. J. Neurosurg..

[11] De Ridder D, Joos K, Vanneste S (2016). Anterior cingulate implants for tinnitus: report of 2 cases. J. Neurosurg..

[12] De Ridder D (2016). Anterior Cingulate Implant for Alcohol Dependence: Case Report. Neurosurgery.

[13] De Ridder D, Leong SL, Manning P, Vanneste S, Glue P (2016). Anterior Cingulate Implant for Obsessive-Compulsive Disorder. World Neurosurg..

[14] Hayward G, Goodwin GM, Harmer CJ (2004). The role of the anterior cingulate cortex in the counting Stroop task. Exp. Brain Res..

[15] Hayward G (2007). Exploring the physiological effects of double-cone coil TMS over the medial frontal cortex on the anterior cingulate cortex: an H2(15)O PET study. Eur. J. Neurosci..

[16] Vanneste S, Ost J, Langguth B, De Ridder D (2014). TMS by double-cone coil prefrontal stimulation for medication resistant chronic depression: a case report. Neurocase.

[17] Modirrousta M, Meek BP, Sareen J, Enns MW (2015). Impaired trial-by-trial adjustment of cognitive control in obsessive compulsive disorder improves after deep repetitive transcranial magnetic stimulation. BMC Neurosci..

[18] Modirrousta M (2015). The efficacy of deep repetitive transcranial magnetic stimulation over the medial prefrontal cortex in obsessive compulsive disorder: results from an open-label study. Depress. Anxiety.

[19] De Ridder D, Vanneste S, Kovacs S, Sunaert S, Dom G (2011). Transient alcohol craving suppression by rTMS of dorsal anterior cingulate: an fMRI and LORETA EEG study. Neurosci. Lett..

[20] Vanneste S, De Ridder D (2013). Differences between a single session and repeated sessions of 1 Hz TMS by double-cone coil prefrontal stimulation for the improvement of tinnitus. Brain Stimul.

[21] Vanneste S, Plazier M, Van de Heyning P, De Ridder D (2011). Repetitive transcranial magnetic stimulation frequency dependent tinnitus improvement by double cone coil prefrontal stimulation. J. Neurol. Neurosurg. Psychiatry.

[22] Naro A, Leo A, Bramanti P, Calabro RS (2015). Moving Toward Conscious Pain Processing Detection in Chronic Disorders of Consciousness: Anterior Cingulate Cortex Neuromodulation. J. Pain.

[23] Tzabazis A (2013). Shaped magnetic field pulses by multi-coil repetitive transcranial magnetic stimulation (rTMS) differentially modulate anterior cingulate cortex responses and pain in volunteers and fibromyalgia patients. Mol. Pain.

[24] Deng ZD, Lisanby SH, Peterchev AV (2014). Coil design considerations for deep transcranial magnetic stimulation. Clin. Neurophysiol..

[25] Deng ZD, Lisanby SH, Peterchev AV (2013). Electric field depth-focality tradeoff in transcranial magnetic stimulation: simulation comparison of 50 coil designs. Brain Stimul.

[26] Rossi S, Hallett M, Rossini PM, Pascual-Leone A (2009). Safety of, T. M. S. C. G. Safety, ethical considerations, and application guidelines for the use of transcranial magnetic stimulation in clinical practice and research. Clin. Neurophysiol..

[27] Wassermann EM (1998). Risk and safety of repetitive transcranial magnetic stimulation: report and suggested guidelines from the International Workshop on the Safety of Repetitive Transcranial Magnetic Stimulation, June 5–7, 1996. Electroencephalogr. Clin. Neurophysiol..

[28] Moreno-Duarte, I. *et al*. In *The Stimulated Brain: Cognitive enhancement using non-invasive brain stimulation* (ed R.C. Kadosh) (Academia Press, 2014).

[29] Nitsche MA, Paulus W (2000). Excitability changes induced in the human motor cortex by weak transcranial direct current stimulation. J. Physiol..

[30] Miranda PC, Lomarev M, Hallett M (2006). Modeling the current distribution during transcranial direct current stimulation. Clin. Neurophysiol..

[31] Bikson M, Datta A, Rahman A, Scaturro J (2010). Electrode montages for tDCS and weak transcranial electrical stimulation: role of “return” electrode’s position and size. Clin. Neurophysiol..

[32] Pirulli C, Fertonani A, Miniussi C (2014). Is neural hyperpolarization by cathodal stimulation always detrimental at the behavioral level?. Front. Behav. Neurosci..

[33] Miniussi C, Harris JA, Ruzzoli M (2013). Modelling non-invasive brain stimulation in cognitive neuroscience. Neurosci. Biobehav. Rev..

[34] Jacobson L, Koslowsky M, Lavidor M (2012). tDCS polarity effects in motor and cognitive domains: a meta-analytical review. Exp. Brain Res..

[35] Nozari N, Woodard K, Thompson-Schill SL (2014). Consequences of cathodal stimulation for behavior: when does it help and when does it hurt performance?. PLoS One.

[36] DaSilva AF (2015). State-of-art neuroanatomical target analysis of high-definition and conventional tDCS montages used for migraine and pain control. Front. Neuroanat..

[37] Datta, A. *et al*. Gyri-precise head model of transcranial direct current stimulation: improved spatial focality using a ring electrode versus conventional rectangular pad. *Brain Stimul* **2**, 201–207, 207 e201, 10.1016/j.brs.2009.03.005 (2009). 10.1016/j.brs.2009.03.005 PMC2790295 20648973

[38] Dmochowski JP, Datta A, Bikson M, Su Y, Parra LC (2011). Optimized multi-electrode stimulation increases focality and intensity at target. Journal of neural engineering.

[39] Guleyupoglu B, Schestatsky P, Edwards D, Fregni F, Bikson M (2013). Classification of methods in transcranial electrical stimulation (tES) and evolving strategy from historical approaches to contemporary innovations. J. Neurosci. Methods.

[40] Shekhawat GS (2015). Intensity, Duration, and Location of High-Definition Transcranial Direct Current Stimulation for Tinnitus Relief. Neurorehabil. Neural Repair.

[41] Villamar, M. F. *et al*. Technique and considerations in the use of 4x1 ring high-definition transcranial direct current stimulation (HD-tDCS). *Journal of visualized experiments: JoVE*, e50309, 10.3791/50309 (2013). 10.3791/50309 PMC3735368 23893039

[42] To WT, Hart J, De Ridder D, Vanneste S (2016). Considering the influence of stimulation parameters on the effect of conventional and high-definition transcranial direct current stimulation. Expert Rev. Med. Devices.

[43] Donnell A (2015). High-Definition and Non-invasive Brain Modulation of Pain and Motor Dysfunction in Chronic TMD. Brain Stimul.

[44] Villamar MF (2013). Focal modulation of the primary motor cortex in fibromyalgia using 4x1-ring high-definition transcranial direct current stimulation (HD-tDCS): immediate and delayed analgesic effects of cathodal and anodal stimulation. J. Pain.

[45] Borckardt JJ (2012). A pilot study of the tolerability and effects of high-definition transcranial direct current stimulation (HD-tDCS) on pain perception. J. Pain.

[46] Mansouri FA, Tanaka K, Buckley MJ (2009). Conflict-induced behavioural adjustment: a clue to the executive functions of the prefrontal cortex. Nat. Rev. Neurosci..

[47] Bush G, Luu P, Posner MI (2000). Cognitive and emotional influences in anterior cingulate cortex. Trends Cogn. Sci..

[48] Bush G (1998). The counting Stroop: an interference task specialized for functional neuroimaging–validation study with functional MRI. Hum. Brain Mapp..

[49] Whalen PJ (1998). The emotional counting Stroop paradigm: a functional magnetic resonance imaging probe of the anterior cingulate affective division. Biol. Psychiatry.

[50] Bush G (1999). Anterior cingulate cortex dysfunction in attention-deficit/hyperactivity disorder revealed by fMRI and the Counting Stroop. Biol. Psychiatry.

[51] Brown LT (2016). Dorsal anterior cingulotomy and anterior capsulotomy for severe, refractory obsessive-compulsive disorder: a systematic review of observational studies. J. Neurosurg..

[52] Brunoni AR (2011). A systematic review on reporting and assessment of adverse effects associated with transcranial direct current stimulation. Int. J. Neuropsychopharmacol..

[53] Oldfield RC (1971). The assessment and analysis of handedness: the Edinburgh inventory. Neuropsychologia.

[54] Kuo HI (2013). Comparing cortical plasticity induced by conventional and high-definition 4 x 1 ring tDCS: a neurophysiological study. Brain Stimul.

[55] Edwards D (2013). Physiological and modeling evidence for focal transcranial electrical brain stimulation in humans: a basis for high-definition tDCS. Neuroimage.

[56] Caparelli-Daquer EM (2012). A pilot study on effects of 4x1 high-definition tDCS on motor cortex excitability. Conf. Proc. IEEE Eng. Med. Biol. Soc..

[57] Brunoni AR (2012). Clinical research with transcranial direct current stimulation (tDCS): Challenges and future directions. Brain Stimulation.

[58] Nitsche MA (2007). Shaping the effects of transcranial direct current stimulation of the human motor cortex. J. Neurophysiol..

[59] Faria P, Hallett M, Miranda PC (2011). A finite element analysis of the effect of electrode area and inter-electrode distance on the spatial distribution of the current density in tDCS. Journal of neural engineering.

[60] Shekhawat GS, Stinear CM, Searchfield GD (2013). Transcranial direct current stimulation intensity and duration effects on tinnitus suppression. Neurorehabil. Neural Repair.

[61] Datta A, Truong D, Minhas P, Parra LC, Bikson M (2012). Inter-Individual Variation during Transcranial Direct Current Stimulation and Normalization of Dose Using MRI-Derived Computational Models. Front Psychiatry.

[62] Dresler T, Meriau K, Heekeren HR, van der Meer E (2009). Emotional Stroop task: effect of word arousal and subject anxiety on emotional interference. Psychol. Res..

[63] Spielberger, C. D., Gorsuch, R. L., Lushene, R., Vagg, P. R. & Jacobs, G. A. *Manual for the State- Trait Anxiety Inventory*. (Consulting Psychologists Press, 1983).

[64] Tombaugh TNTM, Test A (2004). and B: normative data stratified by age and education. Arch. Clin. Neuropsychol..

[65] Volkow ND (2000). Association between age-related decline in brain dopamine activity and impairment in frontal and cingulate metabolism. The American journal of psychiatry.

[66] Logan JM, Sanders AL, Snyder AZ, Morris JC, Buckner RL (2002). Under-recruitment and nonselective recruitment: dissociable neural mechanisms associated with aging. Neuron.

[67] Siepmann M, Kirch W (2002). Effects of caffeine on topographic quantitative EEG. Neuropsychobiology.

[68] Moazami-Goudarzi M, Michels L, Weisz N, Jeanmonod D (2010). Temporo-insular enhancement of EEG low and high frequencies in patients with chronic tinnitus. QEEG study of chronic tinnitus patients. BMC neuroscience.

[69] Fuchs M, Kastner J, Wagner M, Hawes S, Ebersole JS (2002). A standardized boundary element method volume conductor model. Clin. Neurophysiol..

[70] Jurcak V, Tsuzuki D, Dan I (2007). 10/20, 10/10, and 10/5 systems revisited: their validity as relative head-surface-based positioning systems. Neuroimage.

[71] Vitacco D, Brandeis D, Pascual-Marqui R, Martin E (2002). Correspondence of event-related potential tomography and functional magnetic resonance imaging during language processing. Hum. Brain Mapp..

[72] Mulert C (2004). Integration of fMRI and simultaneous EEG: towards a comprehensive understanding of localization and time-course of brain activity in target detection. Neuroimage.

[73] Worrell GA (2000). Localization of the epileptic focus by low-resolution electromagnetic tomography in patients with a lesion demonstrated by MRI. Brain Topogr..

[74] Dierks T (2000). Spatial pattern of cerebral glucose metabolism (PET) correlates with localization of intracerebral EEG-generators in Alzheimer’s disease. Clin. Neurophysiol..

[75] Pizzagalli DA (2004). Functional but not structural subgenual prefrontal cortex abnormalities in melancholia. Mol. Psychiatry.

[76] Zumsteg D, Wennberg RA, Treyer V, Buck A, Wieser HG (2005). H2(15)O or 13NH3 PET and electromagnetic tomography (LORETA) during partial status epilepticus. Neurology.

[77] Zumsteg D, Lozano AM, Wennberg RA (2006). Depth electrode recorded cerebral responses with deep brain stimulation of the anterior thalamus for epilepsy. Clin. Neurophysiol..

[78] Zumsteg D, Lozano AM, Wieser HG, Wennberg RA (2006). Cortical activation with deep brain stimulation of the anterior thalamus for epilepsy. Clin. Neurophysiol..

[79] Volpe U (2007). The cortical generators of P3a and P3b: a LORETA study. Brain Res. Bull..

[80] Pizzagalli D (2001). Anterior cingulate activity as a predictor of degree of treatment response in major depression: evidence from brain electrical tomography analysis. Am. J. Psychiatry.

[81] Zumsteg D, Lozano AM, Wennberg RA (2006). Mesial temporal inhibition in a patient with deep brain stimulation of the anterior thalamus for epilepsy. Epilepsia.

[82] Mazziotta J (2001). A probabilistic atlas and reference system for the human brain: International Consortium forBrain Mapping (ICBM). Philos. Trans. R. Soc. Lond. B Biol. Sci..

[83] Lancaster JL (2000). Automated Talairach atlas labels for functional brain mapping. Hum. Brain Mapp..

[84] Oostenveld R, Praamstra P (2001). The five percent electrode system for high-resolution EEG and ERP measurements. Clin. Neurophysiol..

[85] Brett M, Johnsrude IS, Owen AM (2002). The problem of functional localization in the human brain. Nat. Rev. Neurosci..

[86] Azizian A (2010). Smoking reduces conflict-related anterior cingulate activity in abstinent cigarette smokers performing a Stroop task. Neuropsychopharmacology.

[87] Froeliger B, Modlin L, Wang L, Kozink RV, McClernon FJ (2012). Nicotine withdrawal modulates frontal brain function during an affective Stroop task. Psychopharmacology (Berl.).

[88] Froeliger B (2013). Frontoparietal attentional network activation differs between smokers and nonsmokers during affective cognition. Psychiatry Res..

[89] Pascual-Marqui RD (2002). Standardized low-resolution brain electromagnetic tomography (sLORETA): technical details. Methods Find. Exp. Clin. Pharmacol..

[90] Nichols TE, Holmes AP (2002). Nonparametric permutation tests for functional neuroimaging: a primer with examples. Hum Brain Mapp.

[91] Whalen PJ, Bush G, Shin LM, Rauch SL (2006). The emotional counting Stroop: a task for assessing emotional interference during brain imaging. Nat. Protoc..

[92] Beissner F, Meissner K, Bar KJ, Napadow V (2013). The autonomic brain: an activation likelihood estimation meta-analysis for central processing of autonomic function. J. Neurosci..

[93] Legrain V, Iannetti GD, Plaghki L, Mouraux A (2011). The pain matrix reloaded: a salience detection system for the body. Prog. Neurobiol..

[94] Miller BL (2001). Neuroanatomy of the self: evidence from patients with frontotemporal dementia. Neurology.

[95] Hsu TY (2011). Modulating inhibitory control with direct current stimulation of the superior medial frontal cortex. Neuroimage.

[96] Kwon YH, Kwon JW (2013). Response Inhibition Induced in the Stop-signal Task by Transcranial Direct Current Stimulation of the Pre-supplementary Motor Area and Primary Sensoriomotor Cortex. J Phys Ther Sci.

[97] D’Urso G (2016). Polarity-dependent effects of transcranial direct current stimulation in obsessive-compulsive disorder. Neurocase.

[98] D’Urso G (2016). Transcranial direct current stimulation for obsessive-compulsive disorder: A randomized, controlled, partial crossover trial. Depress. Anxiety.

[99] Watanabe T (2015). Effects of rTMS of pre-supplementary motor area on fronto basal ganglia network activity during stop-signal task. J. Neurosci..

[100] Obeso I (2013). Stimulation of the pre-SMA influences cerebral blood flow in frontal areas involved with inhibitory control of action. Brain Stimul.

[101] Davis KD (2005). Human anterior cingulate cortex neurons encode cognitive and emotional demands. J. Neurosci..

[102] Schutter DJ, Van Honk J (2005). Electrophysiological ratio markers for the balance between reward and punishment. Brain Res Cogn Brain Res.

[103] Putman P, van Peer J, Maimari I, van der Werff S (2010). EEG theta/beta ratio in relation to fear-modulated response-inhibition, attentional control, and affective traits. Biol Psychol.

[104] Putman P (2011). Resting state EEG delta-beta coherence in relation to anxiety, behavioral inhibition, and selective attentional processing of threatening stimuli. Int J Psychophysiol.

[105] Schack B, Chen ACN, Mescha S, Witte H (1999). Instantaneous EEG coherence analysis during the Stroop task. Clin Neurophysiol.

[106] Song M, Shin Y, Yun K (2014). Beta-frequency EEG activity increased during transcranial direct current stimulation. Neuroreport.

[107] Thibaut A (2017). Neural signature of tDCS, tPCS and their combination: Comparing the effects on neural plasticity. Neurosci Lett.

[108] Inzlicht M, Bartholow BD, Hirsh JB (2015). Emotional foundations of cognitive control. Trends Cogn Sci.

[109] Soutschek A, Muller HJ, Schubert T (2013). Conflict-specific effects of accessory stimuli on cognitive control in the Stroop task and the Simon task. Exp Psychol.

[110] West R (2003). Neural correlates of cognitive control and conflict detection in the Stroop and digit-location tasks. Neuropsychologia.

[111] Luo Q (2013). Theta band activity in response to emotional expressions and its relationship with gamma band activity as revealed by MEG and advanced beamformer source imaging. Frontiers in human neuroscience.

[112] Luu P, Tucker DM (2001). Regulating action: alternating activation of midline frontal and motor cortical networks. Clinical neurophysiology: official journal of the International Federation of Clinical Neurophysiology.

[113] Makeig S (2004). Electroencephalographic brain dynamics following manually responded visual targets. PLoS Biol.

[114] Guo Q (2017). Single-trial EEG-informed fMRI analysis of emotional decision problems in hot executive function. Brain Behav.

[115] Mancini M (2016). Assessing cortical synchronization during transcranial direct current stimulation: A graph-theoretical analysis. Neuroimage.

[116] Ardolino G, Bossi B, Barbieri S, Priori A (2005). Non-synaptic mechanisms underlie the after-effects of cathodal transcutaneous direct current stimulation of the human brain. J Physiol.

[117] Creutzfeldt OD, Fromm GH, Kapp H (1962). Influence of transcortical d-c currents on cortical neuronal activity. Exp Neurol.

